# An epithelial–mesenchymal transition-related mRNA signature associated with the prognosis, immune infiltration and therapeutic response of colon adenocarcinoma

**DOI:** 10.3389/pore.2023.1611016

**Published:** 2023-02-24

**Authors:** Yu Zhang, Yan Li, Zan Zuo, Ting Li, Ying An, Wenjing Zhang

**Affiliations:** ^1^ Department of Gastroenterology, The First People’s Hospital of Yunnan Province, Affiliated Hospital of Kunming University of Science and Technology, Kunming, China; ^2^ Yunnan Digestive Endoscopy Clinical Medical Center, Kunming, China; ^3^ Faculty of Medicine, Kunming University of Science and Technology, Kunming, China; ^4^ Department of Medical Oncology, The First People’s Hospital of Yunnan Province, Affiliated Hospital of Kunming University of Science and Technology, Kunming, China

**Keywords:** epithelial-mesenchymal transition, bioinformatics, prognostic signature, tumor immune microenvironment, colon adenocarcinoma

## Abstract

**Background:** Epithelial-mesenchymal transition (EMT) is closely associated with cancer cell metastasis. Colon adenocarcinoma (COAD) is one of the most common malignancies in the world, and its metastasis leading to poor prognosis remains a challenge for clinicians. The purpose of this study was to explore the prognostic value of EMT-related genes (EMTRGs) by bioinformatics analysis and to develop a new EMTRGs prognostic signature for COAD.

**Methods:** The TCGA-COAD dataset was downloaded from the TCGA portal as the training cohort, and the GSE17538 and GSE29621 datasets were obtained from the GEO database as the validation cohort. The best EMTRGs prognostic signature was constructed by differential expression analysis, Cox, and LASSO regression analysis. Gene set enrichment analysis (GSEA) is used to reveal pathways that are enriched in high-risk and low-risk groups. Differences in tumor immune cell levels were analyzed using microenvironmental cell population counter and single sample gene set enrichment analysis. Subclass mapping analysis and Genomics of Drug Sensitivity in Cancer were applied for prediction of immunotherapy response and chemotherapy response, respectively.

**Results:** A total of 77 differentially expressed EMTRGs were identified in the TCGA-COAD cohort, and they were significantly associated with functions and pathways related to cancer cell metastasis, proliferation, and apoptosis. We constructed EMTRGs prognostic signature with COMP, MYL9, PCOLCE2, SCG2, and TIMP1 as new COAD prognostic biomarkers. The high-risk group had a poorer prognosis with enhanced immune cell infiltration. The GSEA demonstrated that the high-risk group was involved in “ECM Receptor Interaction,” “WNT Signaling Pathway” and “Colorectal Cancer.” Furthermore, patients with high risk scores may respond to anti-CTLA4 therapy and may be more resistant to targeted therapy agents BI 2536 and ABT-888.

**Conclusion:** Together, we developed a new EMTRGs prognostic signature that can be an independent prognostic factor for COAD. This study has guiding implications for individualized counseling and treatment of COAD patients.

## Introduction

Colon adenocarcinoma (COAD), as one of the common gastrointestinal malignancies, has the top incidence and mortality rate among common cancers worldwide. According to the Global Cancer Statistics report published in 2020 (1), colorectal cancer (CRC) has the third highest incidence and the second highest mortality rate of all malignancies. The mortality and morbidity rate of COAD in China is also increasing year by year, which has posed a serious threat to the health of the residents, and caused a heavy burden on the families of patients financially (2). Early-stage COAD can be treated well with surgical resection or with radiotherapy. However, due to the insidious onset of COAD, most patients are diagnosed at the medium or advanced stage with tumor infiltration and metastasis. Notably, approximately 90% of cancer-related mortality is caused by cancer metastasis (3). Consequently, an in-depth exploration of biomarkers in the development and metastasis of COAD will help to establish new diagnostic and therapeutic approaches for COAD.

Epithelial-mesenchymal transition (EMT) is considered to be one of the main mechanisms determining the spread of infiltrative and metastatic cancer cells, a dynamic and reversible process of increased motility and invasiveness of cancer cells. During EMT, epithelial cells gradually lose intercellular adhesion and apical-basal polarity, thus transforming into mesenchymal cells with migratory and invasive abilities (4). On the one hand, EMT plays an important role in the generation of neural crest delamination, gastrula, and a variety of cell and tissue types (4, 5). On the other hand, for cancer cells to acquire an invasive phenotype for metastasis, EMT is aberrantly activated in cancer cells and facilitates their spread from the primary tumor into the circulation, leading to enhanced cell stemness and immune resistance of tumor cells to resist various therapeutic attacks (4, 6). The role of EMT in COAD metastasis has been well demonstrated. Wang et al. (7) showed that Cinobufacini can inhibit EMT to restrain invasion and metastasis of COAD by suppressing Wnt/β-catenin signaling pathway activation *in vivo* and *in vitro*. ACLY can stabilize β-catenin by mutual interaction thus promoting nuclear translocation of β-catenin, which contributes to the EMT process exacerbating COAD metastasis (8). In recent years, with the continuous exploitation of sequencing data, the development of EMT-related genes (EMTRG) prognostic signatures based on public datasets such as TCGA and GEO has been well studied in a variety of malignancies including endometrial cancer (9), bladder cancer (BC) (10, 11), hepatocellular carcinoma (HCC) (12), and pancreatic cancer (13, 14). Nevertheless, the EMTRG prognostic signature in COAD remains to be investigated in depth.

Based on this, we propose to search for new EMTRG prognostic signatures of COAD through public data in TCGA and GEO databases. The aim is to provide potential therapeutic targets and new insights into the mechanisms and functions of EMT in the development of COAD and to explore new prognostic biomarkers for the diagnosis and treatment of metastases in COAD patients.

## Materials and methods

### Data source

The COAD-related data used in this study were obtained from the freely available TCGA (https://portal.gdc.cancer.gov/) and GEO (https://www.ncbi.nlm.nih.gov/geo/) databases. We obtained RNA-seq sequencing data and clinical data of COAD from the TCGA database. The mRNA and lncRNA expression matrices were obtained for 163 TCGA-COAD and 10 normal samples, and miRNA expression profiles were achieved for 158 TCGA-COAD and 3 normal samples. Of the 163 TCGA-COAD samples, those with incomplete survival information and missing clinical data were excluded, and the remaining 154 TCGA-COAD samples were used as the training cohort for screening prognostic genes and evaluating prognostic models in this study.

A total of five COAD-related datasets were downloaded through the GEO database, namely GSE17538, GSE29621, GSE39582, GSE44076, and GSE74602. Among them, the GSE17538 (*n* = 232) and GSE29621 (*n* = 65) datasets containing complete survival information of COAD patients were used as independent external validation cohorts for the validation of the constructed prognostic models. The GSE39582 (566 COAD and 19 normal samples), GSE44076 (98 COAD samples and 148 normal samples), and GSE74602 (30 COAD and 30 normal samples) datasets were used to validate the prognostic gene expression.

The clinical characteristics of COAD patients in the TCGA cohort, GSE17538 dataset, GSE29621 dataset, GSE39582 dataset, GSE44076 dataset, and GSE74602 dataset were shown in Supplementary Table S1.

### Differential expression analysis

Differential expression analysis of mRNA, miRNA, and lncRNA in COAD and normal samples were performed using R package limma. Significance thresholds were set to adjust (adj.) *p* < 0.05 and |log_2_ fold change (FC)| > 1. *P*-values were corrected for multiple testing using the Benjamini & Hochberg method.

The 200 EMTRGs were obtained from the Molecular Signatures Database (MSigDB; http://www.gsea-msigdb.org). Briefly, genes retrieved in MSigDB using the keyword “Epithelial-mesenchymal transition” were defined as EMTRGs. The overlapping genes of EMTRGs and differentially expressed mRNAs (DE-mRNAs), defined as DE-EMTRGs (Supplementary Table S2), were obtained by intersection analysis. Intersection analysis was performed using the Jvenn online tool (http://jvenn.toulouse.inra.fr/app/example.html).

### Construction and confirmation of an EMTRGs prognostic signature

The training cohort containing complete clinical information was first analyzed using univariate Cox regression to select prognostically relevant DE-EMTRGs. After initial screening, a LASSO analysis was established to select candidate DE-EMTRGs with a penalty parameter tuning adjusted by 20 times cross-validation, then a signature based on these well-selected DE-EMTRGs was developed. These prognostically relevant EMTRGs were analyzed by multivariate Cox regression analysis to calculate their respective expression levels and regression coefficients to obtain risk scores. The risk scores were calculated as follows:

$$risk\;score=\sum_{n=1}^{n}\left({coef}_{i}\times x_{i}\right)$$

where *coef*_*i*_ denotes the multivariate Cox regression coefficient of the *i*th gene, *x*_*i*_ denotes the relative expression of the *i*th gene, and *n* denotes the number of genes in model. The risk score of each patient was calculated according to this formula, and patients were divided into high-risk and low-risk groups according to the median of the risk scores. Kaplan-Meier (K-M) analysis and log-rank test were used to compare survival differences between the high-risk and low-risk groups. Then, in the R package SURVIVALROC, time-dependent receiver operating characteristic (ROC) analysis was used to calculate the area under the curve (AUC) for 1-, 3-, and 5-year overall survival (OS), and to determine the predictive accuracy of the model. The above method was used to further validate the predictive performance of the EMTRGs prognostic signature in validation cohort (GSE17538 and GSE29621).

### Independent prognostic analysis

To determine whether this prognostic model was significant among other clinical characteristics, all clinicopathological characteristics in the TCGA-COAD dataset (*n* = 154), including age, gender, pathological T stage, pathological N stage, and pathological M stage, were performed with univariate and multivariate Cox regression analyses to identify the independent clinical prognostic factors using the survival package in R with *p* < 0.05 as the threshold for significance.

### Construction and evaluation of the nomogram

The nomogram and calibration curves were created using the rms package in the R software. The time-dependent ROC curves were used to determine the prognostic performance of the nomogram model with R package survival ROC. Calibration curves were plotted to assess the discrimination of the nomogram and the 45° dotted line indicates the optimal prediction. In addition, decision curve analysis (DCA) was performed to evaluate the clinical usefulness and to compare the established nomogram with the separately identified independent prognostic factors.

### Gene set enrichment analysis (GSEA)

To explore the biological signaling pathways, GSEA was performed on the high-risk and low-risk groups of TCGA-COAD samples, respectively. The filtered KEGG gene set (c2.cp.kegg.v7.4.symbols.gmt) was downloaded from MSigDB. GSEA analysis was performed on the downloaded gene sets using GSEA software (v4.0.3) (15). KEGG pathways with significant enrichment results were demonstrated based on NES (net enrichment score) and *P* value. Gene sets with |NES| > 1, NOM *p* < 0.05, and FDR *q* < 0.25 were considered significantly enriched.

### ESTIMATE analysis

The immune score and stromal cell score were calculated by the ESTIMATE package (16) in the R software, thereby quantifying the proportion of immune stromal components in the tumor microenvironment for each sample. The results were expressed in the form of three scores:ImmuneScore, StromalScore, and ESTIMATEScore, which were positively correlated with the proportion of immune, stromal, and the sum of both, respectively, which means that the higher the respective score, the greater the proportion of the corresponding component in the tumor microenvironment. The Wilcoxon test was used to assess the difference between the three scores of the high-risk and low-risk groups in the TCGA-COAD database.

### Infiltration of immune cells

To further understand the composition of the tumor immune microenvironment (TIME) between the high- and low-risk groups in the TCGA database, we used a microenvironment cell population counter (MCP-counter) (17) to quantify the number of immune cells, fibroblasts and epithelial cells per COAD sample according to marker genes. Then single-sample gene set enrichment analysis (ssGSEA) was performed on tumor tissue infiltrating immune cells, and 28 immune cell types were obtained (16, 18, 19). The significant differences in immune cell numbers were identified by the Wilcoxon test. Furthermore, the correlation between prognostic genes and immune cells was analyzed by the Spearman method. The significance threshold was set at |r| > 0.5 and *p* < 0.05.

### Immunotherapy and chemotherapy response prediction

The subclass mapping (SubMap) modules (20) of GenePattern were used to predict the response of all 154 COAD samples to immune checkpoints. The pRRophetic algorithm (21) based on the Genomics of Drug Sensitivity in Cancer (GDSC) pharmacogenomic database (21) was used to predict chemotherapy response per 154 COAD samples. The half-maximal inhibitory concentration (IC_50_) was estimated by ridge regression, and the prediction accuracy was evaluated by 10-fold cross-validation.

### Construction of ceRNA network

The miRNAs were predicted by miRwalk database, and 504 mRNA-miRNA pairs of 5 prognostic genes were predicted. Overlap analysis of the identified DE-miRNAs and predicted miRNAs obtained 84 DE-miRNAs and 154 mRNA-miRNAs pairs. Subsequently, 18,987 lncRNAs of 89 DE-miRNAs were predicted using the lncbaseV2.0 database with a score >0.6. Of these, 133 predicted lncRNAs were previously obtained DE-lncRNAs. Considering the mechanism of ceRNA, we excluded DE-lncRNAs with an opposite trend of prognostic gene expression in COAD, and obtained a total of 32 mRNA-miRNA relationship pairs (5 mRNAs and 29 miRNAs) and 152 miRNA-lncRNA relationship pairs (29 miRNAs and 76 lncRNAs). Based on *r* > 0.3, *p* < 0.05, Pearson correlation analysis screened 169 pairs of lncRNA-mRNA positive regulatory relationships (5 mRNA and 65 lncRNA). Finally, the above relationship pairs were combined to visualize the lncRNA-miRNA-mRNA ceRNA network by Cytoscape software.

### Statistical analysis

All statistical calculations in this study were performed in R software (version 3.6.1). The Cox proportional hazards regression model was used for univariate and multivariate analyses. The log-rank test was used for K-M survival analyses. The AUC was used as an indicator of prognostic accuracy. Wilcoxon test was used for comparing immune cells and IC50 of drugs between the low-risk and high-risk groups. If not otherwise specified, *p* < 0.05 was a statistically significant threshold.

## Result

### Identification of DE-EMTRGs in the TCGA COAD cohort

**FIGURE 1:**
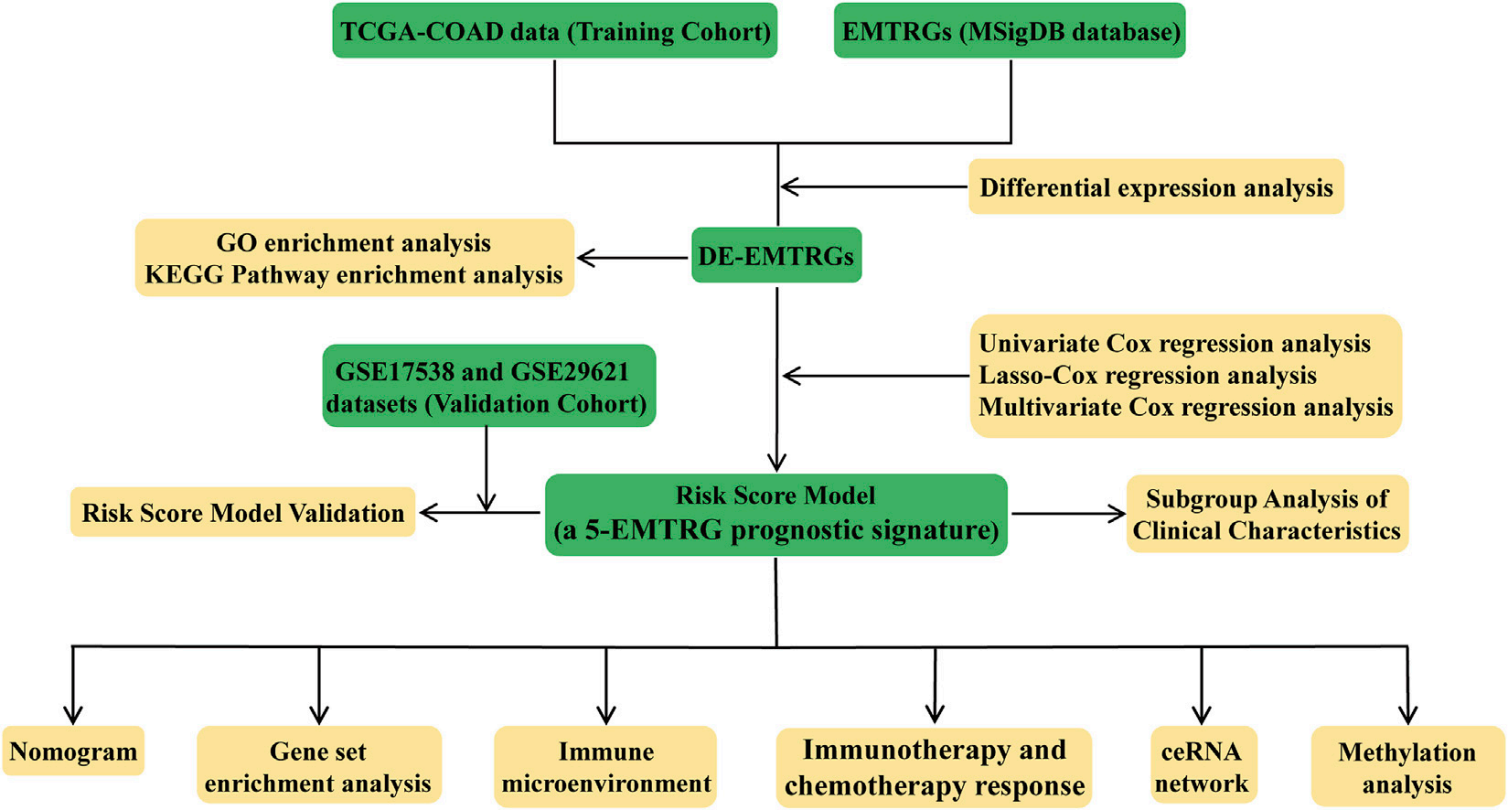
Flow chart of this study.

**FIGURE 2:**
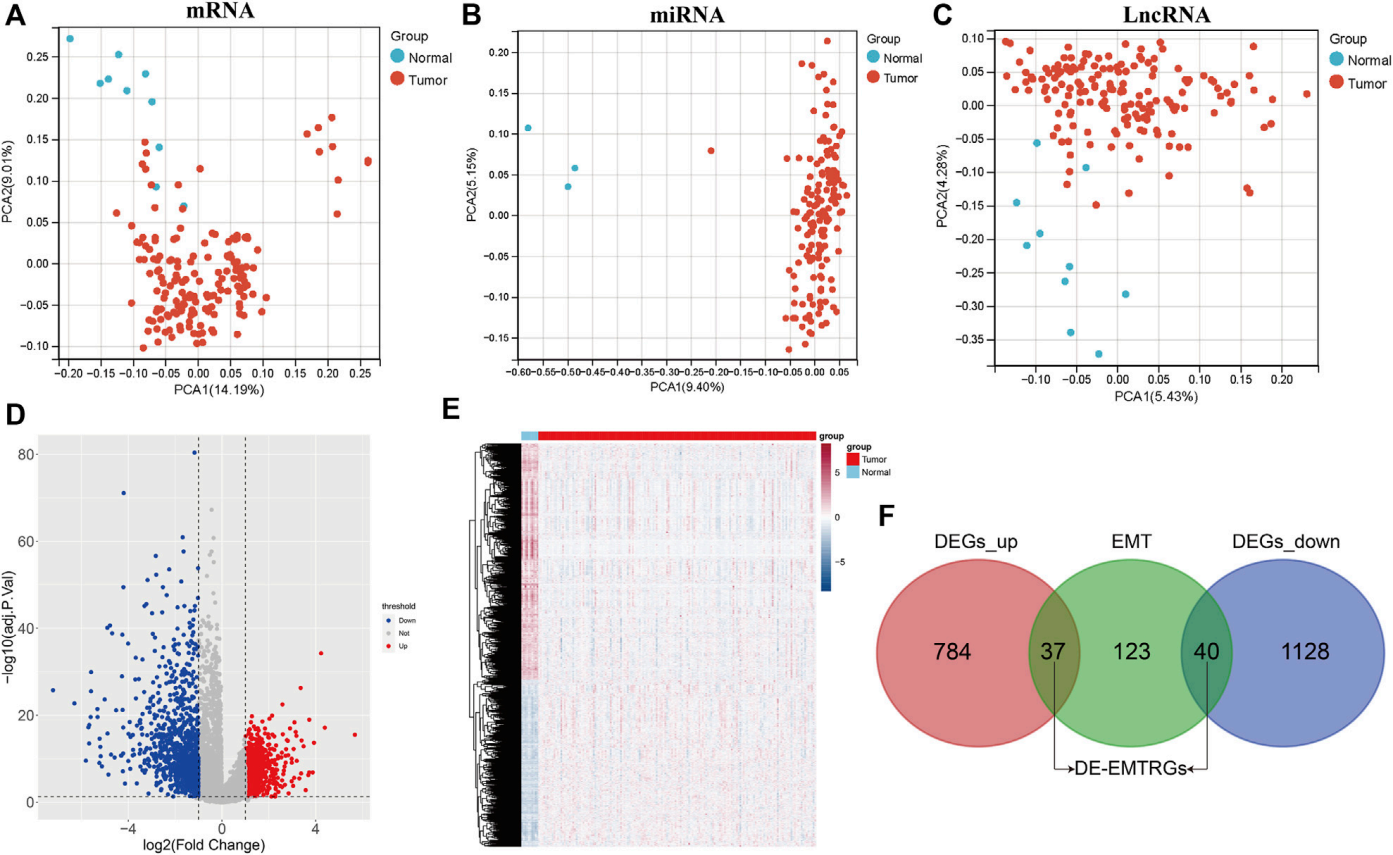
Identification of differentially expressed epithelial-mesenchymal transition related genes (DE-EMTRGs) in COAD. **(A–C)** Principal component analysis reveals differences between expression data of healthy population and COAD samples in TCGA. From left to right are mRNA, miRNA and lncRNA. **(D)** Volcano plot **(E)** and heatmap of differentially expressed mRNA (DE-mRNA) in healthy population and COAD samples in TCGA. **(F)** Venn diagram displayed the number of DE-EMTRGs. Difference thresholds: adj. *p* < 0.05 and |log_2_Fold Change| > 1.

The analysis flow chart of this study is shown in Figure 1. Principal component analysis (PCA) exhibited that the mRNA, miRNA and lncRNA expression data were distributed in different directions in the healthy population and COAD samples (Figures 2A–C). Based on expression data from the TCGA-COAD cohort, a total of 1989 DE-mRNAs containing 1168 down-regulated and 821 up-regulated DE-mRNAs (Figures 2D, E), 585 DE-miRNAs containing 330 down-regulated and 255 upregulated DE-miRNAs (Supplementary Figures S1A, B), and 149 DE-lncRNAs containing 68 downregulated and 81 upregulated DE-lncRNAs (Supplementary Figures S1C, D) were identified. Further, 200 EMTRGs were retrieved through the MSigDB database. Venn diagram displayed the presence of 40 down-regulated and 37 up-regulated DE-EMTRGs in DE-mRNA, respectively (Figure 2F). These DE-EMTRGs are mainly involved in functions and pathways related to cell migration, proliferation and apoptosis such as “negative regulation of cell migration,” “leukocyte migration,” “negative regulation of cell proliferation” and “negative regulation of apoptotic process” (Supplementary Figure S2).

### Establishment and validation of a five-EMTRG prognostic signature for predicting patient-specific survival in COAD

**FIGURE 3:**
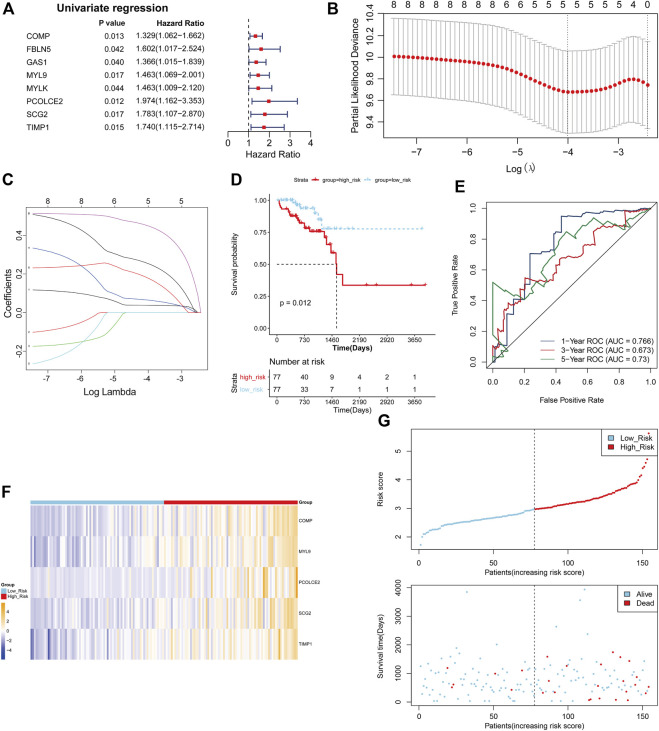
Establishment of a five epithelial-mesenchymal transition-related genes (EMTRGs) prognostic signature for predicting patient-specific survival in COAD. **(A)** Forest plot for univariate Cox regression analysis. **(B)** Penalty maps of the Lasso model for 8 prognostic genes in COAD. The best optimal parameter (λ) was selected by 10-fold cross-validation as 0.01817085. **(C)** LASSO coefficient mapping of 8 prognostic genes. Each curve corresponds to a gene. **(D)** Kaplan-Meier curves indicating the OS in high- and low-risk groups. **(E)** Receiver operating characteristic curves for validating the prognostic performance of risk scores in TCGA training cohort. **(F)** Heatmap of the expression of five-EMTRG prognostic signature in the TCGA training cohort. **(G)** (top) Distribution of risk scores in TCGA training cohort, and (bottom) survival status and time of COAD patients in high- and low-risk groups.

The TCGA COAD cohort was used as the training cohort, and the GSE17538 and GSE29621 datasets were used as the validation cohort to construct and validate the Risk scoring (RS) model for DE-EMTRGs in COAD, respectively. Eight DE-mRNAs associated with survival in COAD patients were identified in 77 DE-EMTRGs by univariate Cox regression analysis (Figure 3A). Lasso regression analysis further screened 5 prognostic biomarkers (COMP, MYL9, PCOLCE2, SCG2, TIMP1; Figures 3B, C). A multivariate Cox analysis was performed based on five prognostic biomarkers, and the coefficient of each biomarker was calculated, and used to construct the RS model. The 154 COAD patients in the training cohort were distinguished by a median risk score of 2.966300771 into high- and low-risk groups containing 77 COAD samples each. K-M curves revealed that OS of COAD patients in the high-risk group was significantly lower than in the low-risk group (*p* = 0.012; Figure 3D). The ROC showed that the AUC of the RS model was 0.766, 0.673 and 0.73 at 1, 3 and 5 years for patients, respectively (Figure 3E), which indicates that this RS has good predictive performance for the prognosis of COAD patients. The expression patterns of the five genes in the high- and low-risk groups are shown in Figure 3F, and COAD patients with higher risk scores had lower survival outcomes (Figure 3G). The GSE17538 and GSE29621 datasets were brought into the RS model for validation, and this five-EMTRG signature has moderate performance for the prognosis of COAD patients with all AUC values greater than 0.6 (Supplementary Figure S3).

COMP and TIMP1 were significantly upregulated, and MYL9, PCOLCE2 and SCG2 were significantly downregulated in TCGA-COAD cohort of the five-EMTRG prognostic signature (Supplementary Figure S4A). In addition, we validated the five-EMTRG prognostic signature in GSE39582, GSE44076 and GSE74602 datasets, and obtained results consistent with the TCGA training cohort (Supplementary Figures S4B–D). Further, we explored the relationship between the RS model and clinicopathological features. The results showed that the risk scores were significantly different at stage I–II and stage III–IV (Supplementary Figure S4G), T1-2 and T3-4 (Supplementary Figure S5H), and N0 and N1-2 (Supplementary Figure S4I), respectively (*p* < 0.05). We also constructed a lncRNA-miRNA-mRNA ceRNA network containing DE-lncRNA and DE-miRNA associated with five-EMTRG prognostic signature. The ceRNA network contained 63 lncRNA-miRNA-mRNA relationship pairs which contained 29 lncRNAs, 18 miRNAs and 4 mRNAs (Supplementary Figure S5).

It is suggested that this five-EMTRG prognostic signature has high specificity and sensitivity for the prediction of survival in COAD, and has good applicability in clinical practice.

### Five-EMTRG prognostic signature is an independent prognostic factor for COAD

**FIGURE 4:**
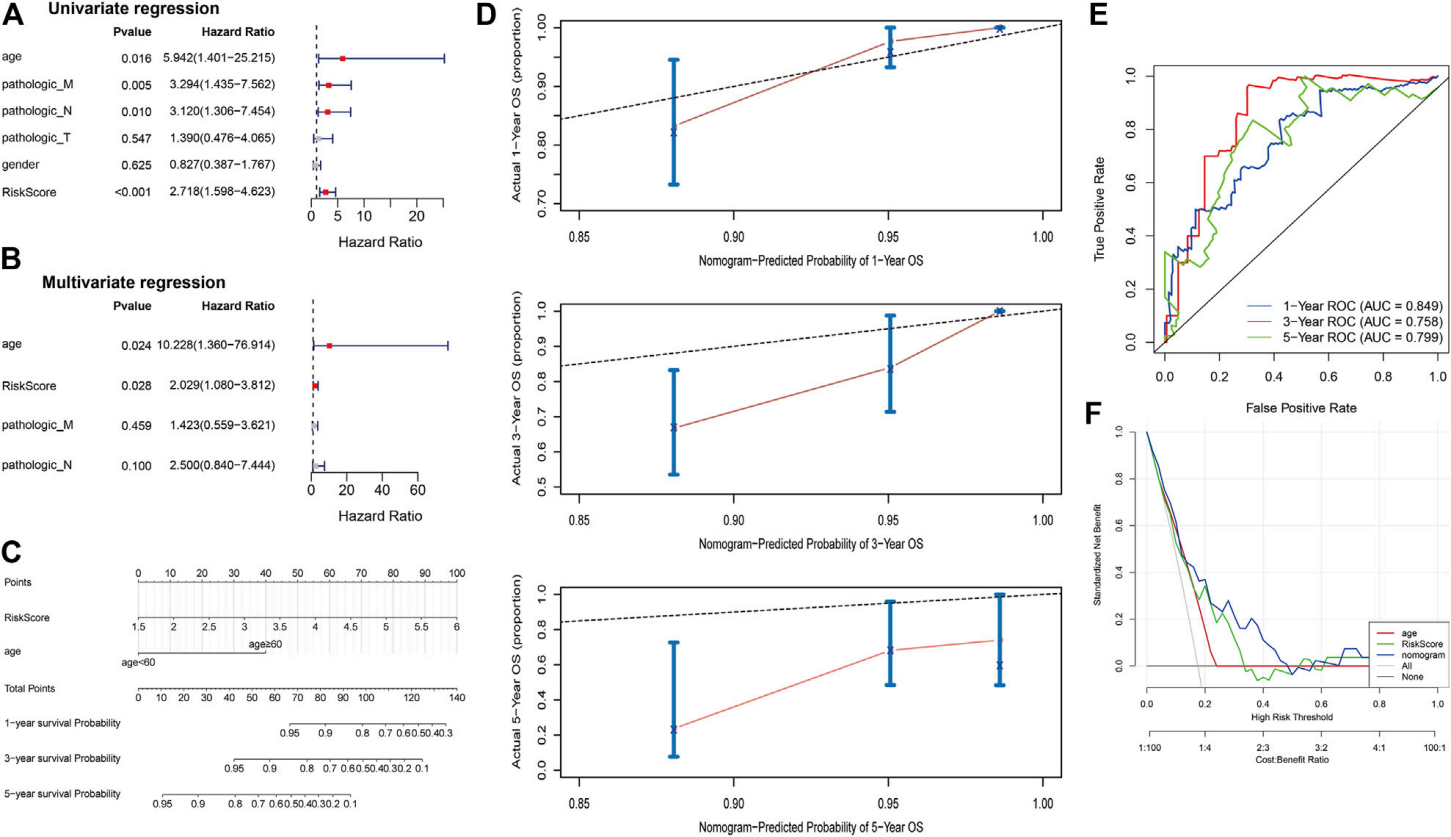
Five epithelial-mesenchymal transition-related genes (EMTRGs) prognostic signature is an independent prognostic factor for COAD. **(A)** Univariate and **(B)** multivariate Cox regression analyses for the identification of independent prognostic factors in the TCGA COAD cohort. **(C)** The nomogram based on independent prognostic factors “age” and “risk score.” **(D)** Calibration plots to assess the accuracy of nomogram predicting (top) 1-, (middle) 3-, and (bottom) 5-year survival rates. **(E)** Receiver operating characteristic curves used to validate the prognostic performance of the nomogram. **(F)** Decision curve analysis for assessing the clinical utility of “nomogram,” “age” and “risk score.”

To explore independent prognostic factors of COAD, we integrated clinicopathological characteristics of the TCGA COAD cohort, including age, gender, pathologic T, pathologic M, and pathologic N, for univariate Cox regression analysis, and the results showed that age (*p* = 0.016), pathologic M (*p* = 0.005), pathologic N (*p* = 0.010), and risk score (*p* < 0.001) were significantly associated with the survival of COAD patients (Figure 4A). Subsequently, the results of univariate Cox regression analysis were enrolled in multivariate Cox regression analysis and demonstrated that age (*p* = 0.024) and risk score (*p* = 0.028) were independent prognostic factors (Figure 4B). Cox regression analyses were also performed in GSE17538 and GSE29621 datasets and found that risk score also was an independent prognostic factor (Supplementary Figures S6A, B). As shown in Figure 4C, we constructed nomogram including age and RS to predict the survival of patients. The calibration curves implied that the 1-year and 3-year patient survival predicted by the nomogram may be similar to the actual survival time, but the 5-year prediction was poor (Figure 4D). The AUC values of 1, 3, and 5 years in the nomogram model were above 0.7, indicating the validity of the constructed nomogram (Figure 4E). The DCA displayed that “nomogram” was higher than the “all,” “age” and “risk score” curves (Figure 4F), indicating that the nomogram model was beneficial within the high-risk threshold range of 0–1, and that the clinical benefit of the nomogram model was higher than the “age” and “risk score” curves. The above results demonstrate that five-EMTRG prognostic signature can be used as an independent prognostic factor for COAD and has the potential for high clinical utility.

### Identification of signaling pathways associated with RS models

**FIGURE 5:**
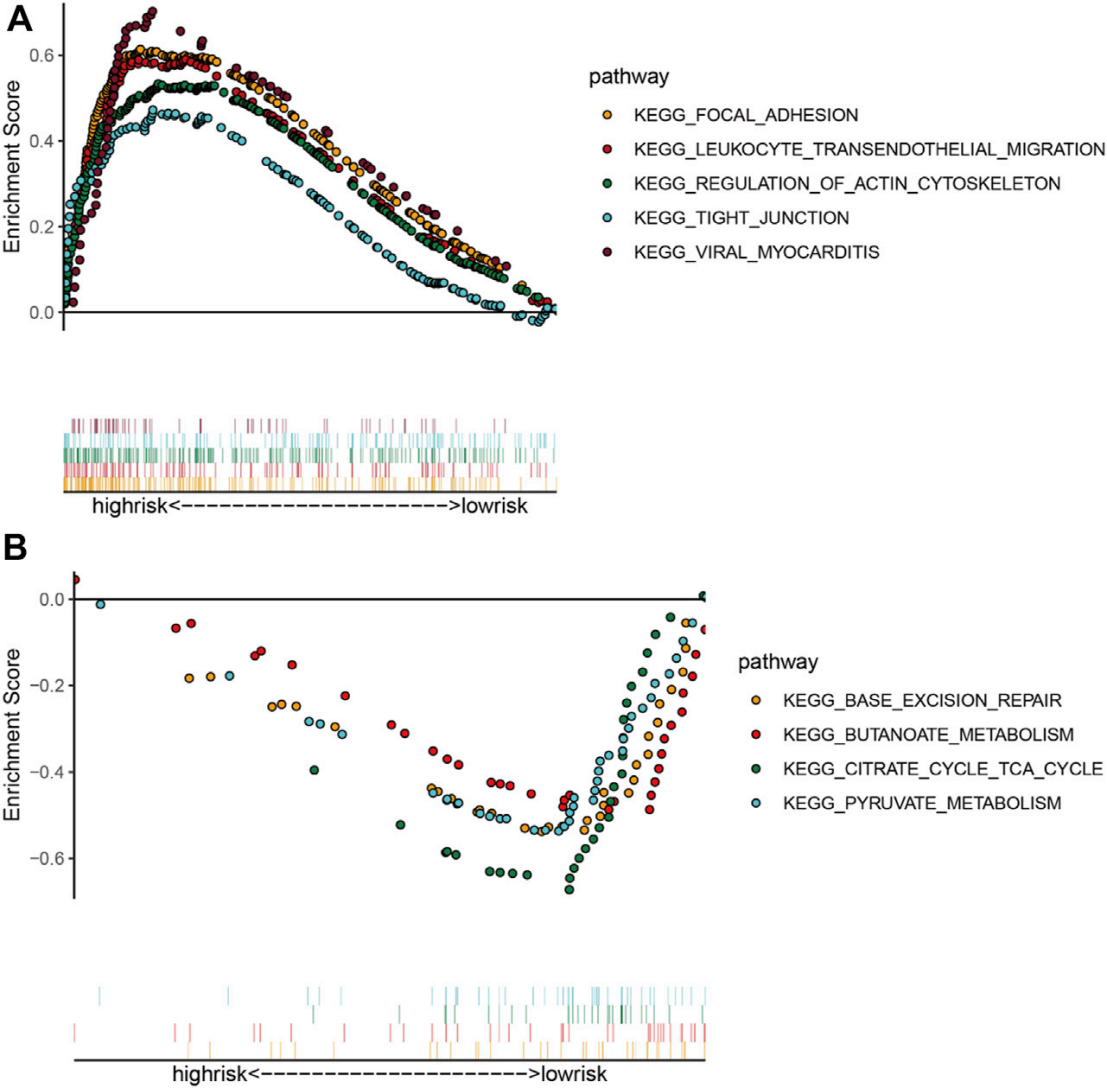
Identification of signaling pathways associated with risk scoring models. The main signaling pathways involved in the **(A)** high- and **(B)** low-risk groups obtained by gene set enrichment analysis. The dots of each color in the upper part of the figure indicate one pathway, and the vertical lines in the lower part indicate the genes covered under each pathway.

Considering the negative correlation between RS and prognosis in COAD patients, we performed GSEA for the high-risk and low-risk groups. The results revealed that the main enriched pathways in the high-risk group include “Focal Adhesion,” “Leukocyte Transendothelial Migration,” “Regulation of Actin Cytoskeleton,” “Tight Junction” and “Viral Myocarditis” (Figure 5A). As expected, classical pathways associated with EMT occurrence (“ECM Receptor Interaction,” “TGF Beta Signaling Pathway,” “WNT Signaling Pathway”) and “Colorectal Cancer” pathway were also significantly enriched in the high-risk group. “Base Excision Repair,” “Butanoate Metabolism,” “Citrate Cycle Tca Cycle” and “Pyruvate Metabolism” were activated mainly in the low-risk group (Figure 5B). Most of the enriched pathways were associated with tumor metastasis, which demonstrated the validity of our five-EMTRG prognostic signature constructed in tumor EMT.

### Correlation of RS model with COAD TIME

**FIGURE 6:**
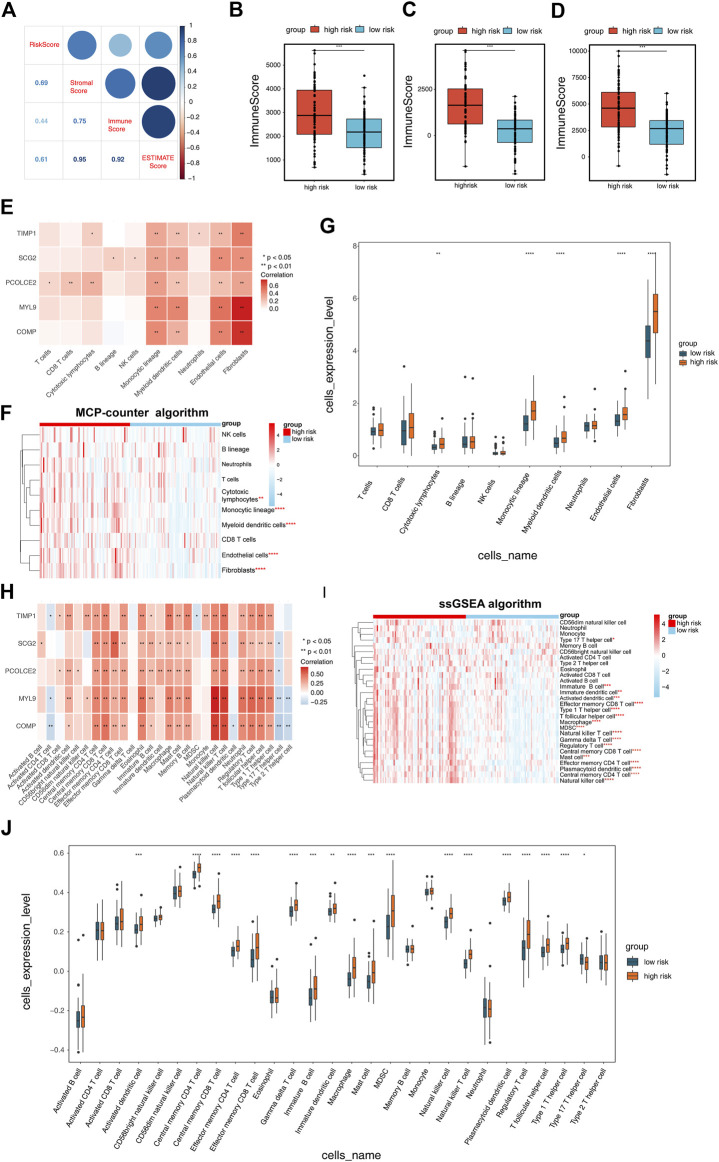
Correlation of risk scoring model with COAD tumor immune microenvironment (TIME). **(A)** The Estimate algorithm was applied to analyze the correlation between risk scores and immune scores, stromal scores, and ESTIMATE scores. Differences in **(B)** ImmuneScores, **(C)** StromalScores and **(D)** ESTIMATEScores of the high and low risk groups obtained from the Estimate algorithm analysis. **(E)** Correlation of risk scoring model with 10 immune cell types was performed by MCP-counter algorithm analysis. **(F)** Heatmap exhibited the enrichment of immune cells in high- and low-risk groups obtained by the MCP-counter algorithm analysis. **(G)** Box plot demonstrated the immune cells with differences in the high- and low-risk groups obtained by the MCP-counter algorithm analysis. **(H)** Correlation of five Epithelial-mesenchymal transition-related genes prognostic signature with 28 immune cell types calculated by single-sample gene set enrichment analysis (ssGSEA) algorithm. **(I)** Heatmap and **(J)** box plot showing the immune cells with differences in the high- and low-risk groups as calculated by the ssGSEA algorithm.

To investigate the relevance of the RS model to the TIME of COAD, we used the Estimate algorithm to evaluate the high- and low-risk groups. The results showed that risk scores were strongly correlated with immune scores, stromal scores, and estimate scores, and presented a positive correlation (Figure 6A). Compared to the high-risk group, the immune score (Figure 6B), stromal score (Figure 6C) and estimate score (Figure 6D) were significantly lower in the low-risk group (*p* < 0.001). To observe the difference of immune cells in the high- and low-risk groups, both MCP-counter algorithm and ssGSEA algorithm were used to infer the abundance of immune cell infiltration, respectively. In the MCP-counter algorithm, COMP exhibited the most significant positive correlation with fibroblasts, MYL9 presented the most significant positive correlation with endothelial cells and fibroblasts, respectively, and TIMP1 displayed the most positive correlation with fibroblasts (Figure 6E). In the ssGSEA algorithm, COMP showed the most significant positive correlation with natural killer cell, natural killer T cell, MYL9 showed the most significantly positively correlated with natural killer cell and natural killer T cell, and SCG2 showed the most significantly positive correlation with effector memory CD4 T cell (Figure 6H). In addition, there were 5 immune cell types that differed between high- and low-risk groups in the MCP-counter algorithm (*p* < 0.01; Figures 6F, G) and 18 in the ssGSEA algorithm (*p* < 0.05; Figures 6I, J).

### Immunotherapy response and targeted therapy agents prediction

**FIGURE 7:**
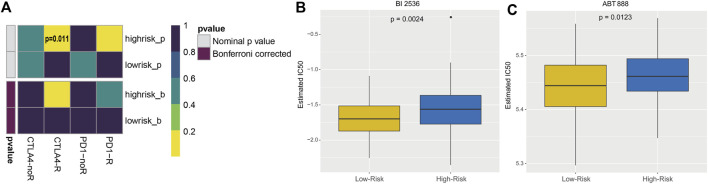
Analysis of risk scoring model-related immunotherapy and chemotherapy prediction. **(A)** Heatmap of the difference in immunotherapy sensitivity between the high- and low-risk groups. Differential IC_50_ of **(B)** BI 2536 and **(C)** ABT-888 in the high- and low-risk groups.

The subclass mapping analysis was used to predict the efficacy of anti-PD1 and anti-CTLA4 treatments. As shown in Figure 7A, we discovered that patient with high risk score may respond to anti-CTLA4 therapy (nominal *p* = 0.011; Figure 7A). Using the pRRophetic algorithm, a ridge regression model was constructed to predict the IC_50_ of targeted therapy agents based on cell line expression profiles and TCGA gene expression profiles from the GDSC database, and the IC_50_ of patients in high- and low-risk groups for these two common targeted therapy agents (BI 2536 and ABT-888) was predicted. The results suggested that patients in the high-risk group may be more resistant to both BI 2536 and ABT-888 compared to the low-risk group (*p* < 0.05; Figures 7B, C). Thus, the 5-EMTRGs prognostic signature could act as a potential predictor for immunotherapies and chemotherapies.

## Discussion

In the present study, we identified 77 DE-EMTRGs in the TCGA-cohort, and they were involved in EMT-related terms, including “negative regulation of cell migration” and “leukocyte migration.” Based on 77 DE-EMTRGs, we developed an RS model consisting of 5 prognostic biomarkers (COMP, MYL9, PCOLCE2, SCG2, TIMP1), and demonstrated that the five-EMTRG prognostic signature can be used as an independent prognostic factor for COAD patients with good clinical utility. We further performed GSEA, and revealed that five-EMTRG prognostic signature was enriched to the pathway associated with tumor metastasis. Furthermore, five-EMTRG prognostic signature correlated with the infiltration abundance of immune cells including Natural killer cells, Natural killer T cells and Effector memory CD4 T cells, and with immune drug CTLA4 inhibitors and targeted therapy agents BI 2536 and ABT-888.

In fact, five biomarkers from the five-EMTRG prognostic signature have been demonstrated to be associated with EMT in a variety of malignancies, including COAD. COMP is an extracellular matrix protein that has been shown to contribute to fibrosis in a variety of visceral organs (22). In CRC, Zhong et al. (23) indicated that COMP is aberrantly highly expressed in CRC tissues, and that it interacts with TAGLN *in vivo* and *in vitro*, leading to cytoskeleton remodeling to promote the EMT process. A bioinformatic analysis showed that COMP is co-expressed with EMT markers in COAD, and is associated with poor patient survival (24). This suggests that COMP contributes to EMT, which also supports the accuracy of the five-EMTRG prognostic signature in COAD. MYL9 is the regulatory light chain that makes up myosin, and its phosphorylation is involved in the tail contraction propulsion of cell migration (25). Previous studies have shown that high expression of MYL9 is associated with poorer prognosis in patients with early-onset CRC (26, 27), epithelial ovarian cancer (28), esophageal squamous cell carcinoma (29) and glioma (30). In contrast to them, Huang et al. (31) showed that downregulation of MYL9 predicted poor biochemical recurrence-free survival in prostate cance, and was significantly associated with prostate cancer cell metastasis. In CRC, MYL9 activates Hippo signaling by binding to YAP1, thereby promoting CRC cell proliferation and metastasis (32). Zhu et al. (33) demonstrated that MYL9 is involved in regulating proliferation and metastasis of CRC stem cells as a downstream of the LncRNA MBNL1-AS1/miR-412-3p axis. PCOLCE2 has been demonstrated to be a prognostically relevant biomarker for CRC (34–36), gastric cancer (37), bladder cancer (38), head and neck squamous cell carcinoma (39), and thyroid cancer (40). However, PCOLCE2 remains to be validated *in vivo* and *in vitro* for its specific mechanism in the EMT process of COAD. SCG2 has been identified as a prognostic biomarker associated with immune infiltration in CRC (41, 42), bladder cancer (43, 44), breast cancer (45), and lung adenocarcinoma (46). Wet assays revealed that SCG2 is lowly expressed in CRC, and inhibits the growth and angiogenesis of CRC cells by promoting the degradation of HIF-1α (47). As an epithelial cell marker, the role of TIMP1 in the tumor EMT process has been well documented (48, 49), and it was identified as a biomarker affecting the prognosis of COAD patients (50). The above study demonstrates the potential and availability of five genes in our five-EMTRG prognostic signature as prognostic biomarkers for COAD.

TME consists mainly of tumor cells, their surrounding tumor-associated fibroblasts (CAFs), inflammatory and immune cells, and non-cellular components including cellular matrix, inflammatory factors and cytokines, and is an extremely complex cellular microenvironment that is considered to be an important factor in tumor development (51, 52). In the course of tumor development, EMT and TIME are mutually crosstalked. It was found that EMT-related genes such as ZEB1 and Snail enrich immunosuppressive cells and inhibit the expression of immunosuppressive molecules through chemokines, leading to the formation of an immunosuppressive microenvironment (53). In turn, immunosuppressive factors lead to tumorigenic EMT (53). The role of TIME in CRC has been well studied. For example, CAFs are abundantly infiltrated with M2 macrophages in CRC, and their markers are poor prognostic factors for CRC (54). Yamila et al. (55) showed that in CRC, the phenotype of natural killer (NK) cells is altered and their receptor expression is drastically reduced, which leads to a reduction in the ability of NK cells to kill cancer cells, and consequently immune escape of tumor cells. In the present study, we found that five-EMTRG prognostic signature was significantly associated with fibroblasts, endothelial cells, NK cells and effector memory CD4 T cells. Notably, the role of the five markers in the five-EMTRG prognostic signature in relation to TIME has been previously reported. For example, COMP correlates with TIME in prostate cancer (56, 57) and bladder cancer (58) and can be a prognostic biomarkers. Zhou et al. (59) revealed by single-cell multi-omics sequencing that MYL9 can serve as a specific biomarker for CAFs, and predicts a poor prognosis for CRC. The nine-gene prognostic signature containing PCOLCE2 constructed by Liu et al. (35) was identified to be associated with CRC TIME. SCG2 was identified as a prognostic biomarker associated with macrophage polarization and immune cell infiltration in CRC (42). Nevertheless, as in the present study, the above-mentioned studies only investigated the correlation between prognostic biomarkers and TIME, but the specific mechanisms of these 5 prognostic biomarkers in EMT and TIME of COAD still need to be further explored.

BI 2536 and ABT-888 are two novel targeted therapy agents that are specific inhibitors of PLK1 and PARP, respectively, and are currently in clinical trials (60, 61). It was shown that BI 2536 effectively impedes mitosis of COAD *in vivo* and *in vitro*, and can synergize with simvastatin for treatment (62) and has a sensitizing effect on radiotherapy (63). ABT-888 has been applied in a phase II clinical trial, and its combination with capecitabine (64), temozolomide (65) and FOLFIRI ± bevacizumab (66) alleviates metastatic colorectal cancer with no unexpected safety concerns. CTLA-4 is a member of the immunoglobulin-associated receptor family, which mediates the suppression of T-cell activation. Ipilimumab (67, 68) and tremelimumab (69,70), which are CTLA4 inhibitors, were effective for improving the survival of patients with metastatic colorectal cancer in phase II clinical trials. The present study found that the high-risk group may be more tolerant to treatment with BI 2536, ABT 888 and CTLA4 inhibitors than the low-risk group in the RS model. Notably, to investigate the possible molecular mechanisms of five biomarkers in the COAD process, we constructed a five-EMTRG prognostic signature-related ceRNA network based on the identified DE-lncRNA and DE-miRNA. The network contains 63 lncRNA-miRNA-mRNA relationship pairs, and some of these DE-lncRNAs and DE-miRNAs have been demonstrated to be involved in COAD. Hsa-miR-16-5p (71, 72), hsa-miR-188-5p (73, 74), lncRNA ADAMTS9-AS1 (75) and LncRNA HAND2-AS1 (76), which are anti-cancer factors, and hsa-miR-192-3p (77), lncRNA MAFG-AS1 (78,79) and lncRNA HAGLR (80), which are pro-cancer factors, are involved in the COAD process by regulating proliferation, apoptosis and EMT phenotype. However, the role of these lncRNA-miRNA-mRNA axes in the COAD process remains to be further validated. It is worth noting that previous studies have identified prognostic expression of signatures associated with EMT in CRC (81–85). In contrast to these studies, only COAD was investigated in this study, and the five-EMTRG prognostic signature was constructed differently from these studies and proved to have a high clinical potential. Furthermore, compared with the studies of Liu (82), Wang (83) and Liao (84) et al., we more comprehensively analyzed the correlation between five-EMTRG prognostic signature and TIME and immunotherapy and chemotherapy response, and constructed the ceRNA network associated with it. Nevertheless, this study has many shortcomings. The sample size of this study is small, including only 163 TCGA-COAD samples from the TCGA-COAD data as the training cohort, and external datasets other than the GEO database are needed for further validation of our model. The lack of *in vivo* and *in vitro* wet experiments to validate the specific regulatory mechanisms of the five biomarkers in the five-EMTRG prognostic signature on the COAD EMT process, which is the focus of our subsequent studies.

In conclusion, we construct a novel five-EMTRG prognostic signature, that can be applied to predict the prognosis of COAD patients, and can be a critical factor in TIME and immunotherapy and chemotherapy. Our results provide greater insight into the role of these key prognostic factors in COAD, and provide a basis for their future use as potential diagnostic and therapeutic biomarkers for COAD.

## Data Availability

The original contributions presented in the study are included in the article/Supplementary Material, further inquiries can be directed to the corresponding author.
