# Supplementary material for: An epithelial–mesenchymal transition-related mRNA signature associated with the prognosis, immune infiltration and therapeutic response of colon adenocarcinoma
Source: Pathol Oncol Res. 2023 Feb 24;29:1611016. doi: 10.3389/pore.2023.1611016 (PMC9998511; doi:10.3389/pore.2023.1611016)
Supplement: Supplementary file 1 [file DataSheet2.docx]

Supplementary Table 1. Clinical characteristics of COAD patients in TCGA cohort, GSE17538 dataset, GSE29621 dataset,GSE39582 dataset, GSE44076 dataset, and GSE74602 dataset.

| **Characteristics** |  | **TCGA**  **(n=154)** | **GSE17538**  **(N=232)** | **GSE29621**  **(n=65)** | **GSE39582**  **(n=566)** | **GSE44076**  **(n=98)** | **GSE74602**  **(n=30)** |
| --- | --- | --- | --- | --- | --- | --- | --- |
| age | age<60 | 46 | 79 | NA | 150 | 12 | NA |
|  | age≥60 | 108 | 153 | NA | 415 | 86 | NA |
|  | unknown |  |  |  | 1 |  |  |
| gender | female | 68 | 110 | 25 | 256 | 27 | NA |
|  | male | 86 | 122 | 40 | 310 | 71 | NA |
| pathologic_M | M0 | 117 | NA | 46 | 482 | NA | NA |
|  | M1 | 22 | NA | 18 | 61 | NA | NA |
|  | unknown | 15 |  | 1 | 23 |  |  |
| pathologic_N | N0 | 78 | NA | 32 | 302 | NA | NA |
|  | N1 | 42 | NA | 25 | 134 | NA | NA |
|  | N2 | 31 | NA | 7 | 98 | NA | NA |
|  | N3 | 0 | NA | 0 | 6 | NA | NA |
|  | unknown | 3 |  | 1 | 26 |  |  |
| pathologic_T | T1 | 8 | NA | 0 | 11 | NA | NA |
|  | T2 | 27 | NA | 8 | 45 | NA | NA |
|  | T3 | 105 | NA | 52 | 367 | NA | NA |
|  | T4 | 13 | NA | 5 | 119 | NA | NA |
|  | unknown | 1 |  |  | 24 |  |  |
| tumor_stage | stage Ⅰ | 28 | 28 | 7 | 33 | 0 | NA |
|  | stage Ⅱ | 47 | 72 | 22 | 264 | 98 | NA |
|  | stage Ⅲ | 47 | 76 | 18 | 205 | 0 | NA |
|  | stage Ⅳ | 23 | 56 | 18 | 60 | 0 | NA |
|  | unknown | 9 |  |  | 4 |  |  |
| Survival state | Alive | 127 | 139 | 40 | 371 | NA | NA |
|  | Dead | 27 | 93 | 25 | 191 | NA | NA |
|  | unknown |  |  |  | 4 |  |  |

In these used datasets, some cases are missed by defining with clinical parameters. Unknown is used to label these missing cases.

NA: Not applicable.

Supplementary Table 2. 77 differentially expressed epithelial-mesenchymal transition related genes.

| **Symbel** | **logFold Change** | **AveExpr** | **t** | **P.Value** | **adjust P** | **B** |
| --- | --- | --- | --- | --- | --- | --- |
| MMP1 | 3.303624997 | 4.308058911 | 5.249184918 | 4.47E-07 | 3.17E-06 | 5.373513893 |
| TGFBI | 3.17970776 | 6.659059629 | 9.513876027 | 1.67E-17 | 5.35E-16 | 28.9757657 |
| MMP3 | 3.08379856 | 3.54820174 | 5.301093419 | 3.50E-07 | 2.53E-06 | 5.609579523 |
| CXCL1 | 2.646185259 | 5.396222561 | 6.158792856 | 5.04E-09 | 5.12E-08 | 9.739458483 |
| CTHRC1 | 2.387804507 | 3.540564628 | 5.317114231 | 3.25E-07 | 2.36E-06 | 5.682774695 |
| COL1A1 | 2.297144659 | 7.328670405 | 4.780572772 | 3.74E-06 | 2.21E-05 | 3.320340778 |
| CXCL8 | 2.293038155 | 4.057171567 | 3.932722087 | 0.000121691 | 0.000514705 | -0.009675954 |
| BGN | 2.187619396 | 6.251718647 | 5.069218646 | 1.03E-06 | 6.79E-06 | 4.568187804 |
| TNFRSF12A | 2.050046943 | 4.799635585 | 8.332886958 | 2.43E-14 | 5.50E-13 | 21.77604456 |
| COMP | 2.029994091 | 2.073597438 | 3.892832508 | 0.000141617 | 0.000588551 | -0.153273326 |
| INHBA | 1.895776501 | 2.086247793 | 5.353685271 | 2.73E-07 | 2.01E-06 | 5.850450512 |
| TIMP1 | 1.82051017 | 7.395179445 | 7.3916155 | 6.08E-12 | 9.80E-11 | 16.33154092 |
| SPP1 | 1.774484901 | 4.307288248 | 2.666726612 | 0.008391555 | 0.022144539 | -3.938487535 |
| COL11A1 | 1.749513483 | 1.78035536 | 4.321820559 | 2.61E-05 | 0.000128375 | 1.454008911 |
| PMEPA1 | 1.702206529 | 5.041701283 | 5.476262597 | 1.52E-07 | 1.18E-06 | 6.418397089 |
| SNTB1 | 1.64214453 | 3.291915392 | 6.305770005 | 2.34E-09 | 2.51E-08 | 10.48766714 |
| VEGFA | 1.57351141 | 3.700073073 | 7.860839614 | 4.02E-13 | 7.82E-12 | 19.00743229 |
| COL7A1 | 1.564246089 | 1.840159928 | 5.190187292 | 5.88E-07 | 4.08E-06 | 5.107249964 |
| PLOD3 | 1.529838644 | 5.275398024 | 8.063265387 | 1.22E-13 | 2.51E-12 | 20.18585842 |
| SERPINE1 | 1.484994374 | 3.200601889 | 3.420804174 | 0.000780211 | 0.002708819 | -1.75760672 |
| MSX1 | 1.46937186 | 1.861074687 | 4.33593579 | 2.47E-05 | 0.000122114 | 1.509209759 |
| SFRP4 | 1.441727649 | 2.781405408 | 2.538307462 | 0.012027895 | 0.030280982 | -4.260726881 |
| LOXL2 | 1.42067745 | 3.424157799 | 4.854380873 | 2.70E-06 | 1.63E-05 | 3.634169757 |
| AREG | 1.336499636 | 5.569541038 | 2.837424651 | 0.00509565 | 0.014343797 | -3.487581554 |
| SERPINH1 | 1.317478356 | 5.415600105 | 6.109916456 | 6.49E-09 | 6.46E-08 | 9.493128767 |
| IGFBP2 | 1.299350585 | 4.591892619 | 2.358052844 | 0.019496474 | 0.045971469 | -4.688076093 |
| MCM7 | 1.274355918 | 5.872557549 | 7.429643495 | 4.90E-12 | 8.03E-11 | 16.545338 |
| MEST | 1.229198085 | 4.097473737 | 6.927218045 | 8.26E-11 | 1.11E-09 | 13.76799872 |
| COL5A1 | 1.203808452 | 4.209493757 | 3.271392201 | 0.001293515 | 0.004243537 | -2.227942352 |
| COL5A2 | 1.146035103 | 4.210299595 | 3.014765916 | 0.002961828 | 0.008857174 | -2.992246579 |
| CD44 | 1.129065941 | 5.007339965 | 6.272703145 | 2.79E-09 | 2.95E-08 | 10.31836978 |
| COL4A1 | 1.12577294 | 5.413090452 | 3.610540902 | 0.000400763 | 0.001495182 | -1.134054787 |
| MMP14 | 1.109536792 | 5.714190514 | 3.633136135 | 0.000369538 | 0.001393189 | -1.057866661 |
| COL1A2 | 1.081631598 | 6.611727048 | 2.430405139 | 0.016110985 | 0.039039527 | -4.520068768 |
| COL12A1 | 1.041034218 | 3.755047134 | 2.725899508 | 0.007077487 | 0.019132802 | -3.78508093 |
| ADAM12 | 1.016344706 | 1.183482006 | 3.429472182 | 0.000757268 | 0.002636594 | -1.729757055 |
| PVR | 1.001420763 | 4.436382888 | 6.340620865 | 1.95E-09 | 2.12E-08 | 10.6666973 |
| GEM | -1.010514699 | 2.958941861 | -3.258272151 | 0.001351136 | 0.00441201 | -2.268360301 |
| FBLN5 | -1.0190001 | 2.010832282 | -3.994091381 | 9.62E-05 | 0.000415804 | 0.213625835 |
| DST | -1.03010906 | 1.672994429 | -5.466498899 | 1.59E-07 | 1.24E-06 | 6.372826037 |
| ID2 | -1.042577674 | 5.167494507 | -4.908707818 | 2.12E-06 | 1.31E-05 | 3.867491674 |
| LAMA1 | -1.050436025 | 0.342449816 | -10.70557531 | 8.20E-21 | 3.62E-19 | 36.52593599 |
| VIM | -1.053294397 | 5.578010827 | -3.435977318 | 0.000740467 | 0.002583572 | -1.7088162 |
| EMP3 | -1.086219135 | 3.474616931 | -4.009448768 | 9.06E-05 | 0.000394004 | 0.269955044 |
| SGCB | -1.191866262 | 2.943743103 | -6.385410447 | 1.54E-09 | 1.71E-08 | 10.89767836 |
| SLIT2 | -1.219063936 | 0.594500564 | -6.964277124 | 6.73E-11 | 9.17E-10 | 13.96922986 |
| FGF2 | -1.229078709 | 0.579286798 | -8.715812085 | 2.38E-15 | 6.05E-14 | 24.07158564 |
| TGFBR3 | -1.334766709 | 1.65048111 | -5.668961225 | 5.96E-08 | 4.99E-07 | 7.329334807 |
| DPYSL3 | -1.498323543 | 3.185043582 | -4.074070882 | 7.05E-05 | 0.000313561 | 0.508938379 |
| FBLN2 | -1.525155501 | 2.90343661 | -3.648632312 | 0.000349465 | 0.001325371 | -1.005380245 |
| FUCA1 | -1.562055905 | 5.319804274 | -8.297834753 | 3.00E-14 | 6.67E-13 | 21.5680388 |
| GAS1 | -1.592220281 | 1.291573361 | -4.543137524 | 1.04E-05 | 5.59E-05 | 2.3359656 |
| PMP22 | -1.642426264 | 3.840365174 | -5.899916509 | 1.89E-08 | 1.72E-07 | 8.449266119 |
| ACTA2 | -1.660021062 | 5.789259573 | -4.260641213 | 3.35E-05 | 0.000160629 | 1.216428447 |
| FAS | -1.702994992 | 2.139490493 | -7.414061255 | 5.35E-12 | 8.73E-11 | 16.45766507 |
| FBLN1 | -1.746124137 | 3.757744606 | -4.176319998 | 4.70E-05 | 0.000217748 | 0.893477266 |
| SLIT3 | -1.805965438 | 0.939455579 | -9.607745765 | 9.25E-18 | 3.02E-16 | 29.56178468 |
| FERMT2 | -1.824999774 | 1.745500749 | -7.132918967 | 2.63E-11 | 3.84E-10 | 14.89242915 |
| TPM1 | -1.986186145 | 4.68881281 | -8.830446219 | 1.18E-15 | 3.09E-14 | 24.76660494 |
| SCG2 | -2.011471038 | 1.083328801 | -8.514892078 | 8.09E-15 | 1.94E-13 | 22.86193889 |
| CAP2 | -2.117764547 | 1.17954263 | -10.62804895 | 1.35E-20 | 5.89E-19 | 36.02849073 |
| MATN2 | -2.148916656 | 2.364320064 | -6.391562503 | 1.49E-09 | 1.66E-08 | 10.92948285 |
| PCOLCE2 | -2.17361762 | 0.461361478 | -14.11674745 | 1.55E-30 | 1.60E-28 | 58.75363754 |
| FLNA | -2.184816965 | 5.895518965 | -5.872756301 | 2.17E-08 | 1.95E-07 | 8.316011509 |
| CALD1 | -2.185453751 | 4.058115027 | -5.55974665 | 1.02E-07 | 8.15E-07 | 6.810368105 |
| EDIL3 | -2.318901871 | 2.240528664 | -9.627278478 | 8.17E-18 | 2.69E-16 | 29.6839416 |
| ABI3BP | -2.35967993 | 0.670210369 | -14.7858604 | 1.94E-32 | 2.43E-30 | 63.10874631 |
| MGP | -2.474019881 | 3.965861763 | -5.487415723 | 1.44E-07 | 1.13E-06 | 6.470522926 |
| DCN | -2.486939786 | 3.678443108 | -6.03779767 | 9.39E-09 | 9.05E-08 | 9.131967041 |
| TPM2 | -2.639363266 | 4.917170367 | -6.786524173 | 1.79E-10 | 2.29E-09 | 13.00956872 |
| CXCL12 | -2.879919008 | 2.116821366 | -11.33652782 | 1.35E-22 | 6.96E-21 | 40.59802568 |
| MFAP5 | -2.900504171 | 1.461405965 | -8.855474037 | 1.01E-15 | 2.67E-14 | 24.91879936 |
| ANPEP | -2.995595862 | 2.921974709 | -7.863622739 | 3.96E-13 | 7.70E-12 | 19.02354036 |
| TAGLN | -3.147919382 | 5.447718317 | -6.826532038 | 1.44E-10 | 1.87E-09 | 13.22433474 |
| MYL9 | -3.311109944 | 5.900102516 | -8.007166495 | 1.70E-13 | 3.44E-12 | 19.85790815 |
| SFRP1 | -3.319786879 | 0.768801688 | -13.23630012 | 5.09E-28 | 4.20E-26 | 52.99880602 |
| MYLK | -3.48956304 | 2.363268768 | -10.60659738 | 1.56E-20 | 6.71E-19 | 35.89097555 |
