# Supplementary material for: An epithelial–mesenchymal transition-related mRNA signature associated with the prognosis, immune infiltration and therapeutic response of colon adenocarcinoma
Source: Pathol Oncol Res. 2023 Feb 24;29:1611016. doi: 10.3389/pore.2023.1611016 (PMC9998511; doi:10.3389/pore.2023.1611016)
Supplement: Supplementary file 2 [file DataSheet1.docx]

**Supplementary figures**

**
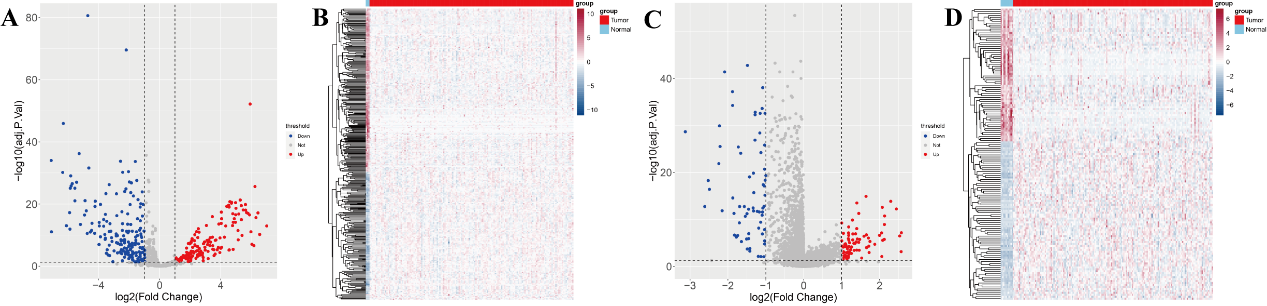
**

**Supplementary Figure 1 Identification of DE-miRNA and DE-lncRNA in TCGA-COAD cohort.** (A) Volcano plot and (B) heatmap of DE-miRNAs between healthy population and COAD samples in TCGA. (C) Volcano plot and (D) heatmap of DE-lncRNAs. Difference thresholds: adj. P < 0.05 and |log2FC| >1.

**
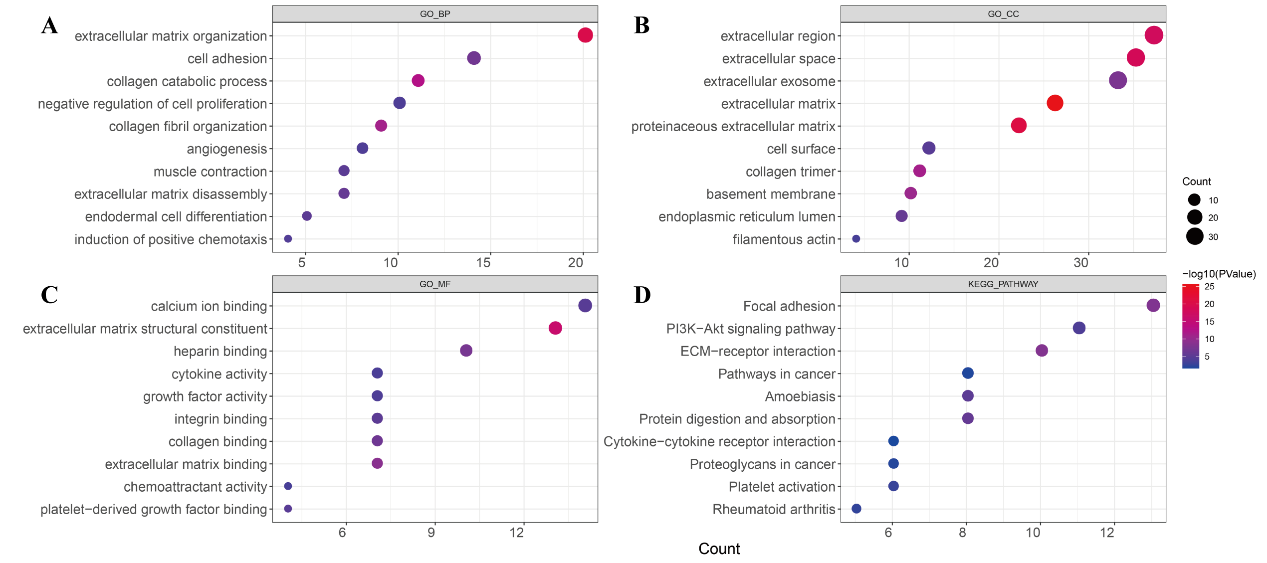
**

**Supplementary Figure 2 GO and KEGG pathway enrichment analysis of DE-EMTRGs.** (A-C) GO and (D) KEGG of DE-EMTRGs were explored using the Database for Annotation, Visualization and Integrated Discovery (DAVID; V6.8; https://david-d.ncifcrf.gov/). The GO results contained three categories: (A) biological processes (BP), (B) cellular components (CC), and (C) molecular functions (MF). Enriched GO terms and KEGG pathways were determined according to the adj. critical criterion P < 0.05 and count ≥ 2.

**
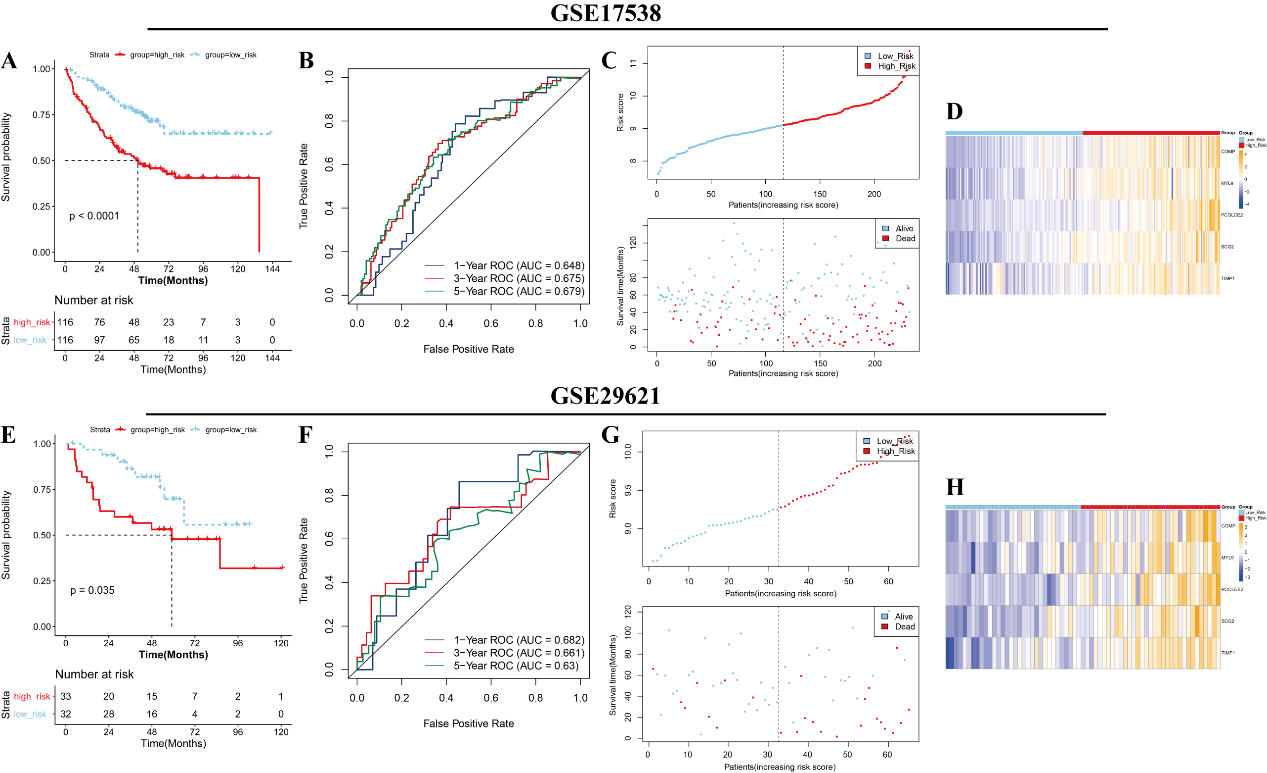
**

**Supplementary Figure 3** **Validation of a five-EMTRG prognostic signature for predicting patient-specific survival in COAD.** (A and E) K-M curves indicated the likelihood of survival for patients in the high and low risk groups of the GSE17538 and GSE29621 datasets. (B and F) Time-related ROC curves were used to validate the prognostic performance of the risk scores in the GSE17538 and GSE29621 datasets. (C and G) (Top) Distribution of risk scores in the GSE17538 and GSE29621 datasets and (Bottom) survival status and survival time of patients with COAD in the high and low risk groups. (D and H) Heatmap of the expression of five-EMTRG prognostic signature in GSE17538 and GSE29621 datasets.

**
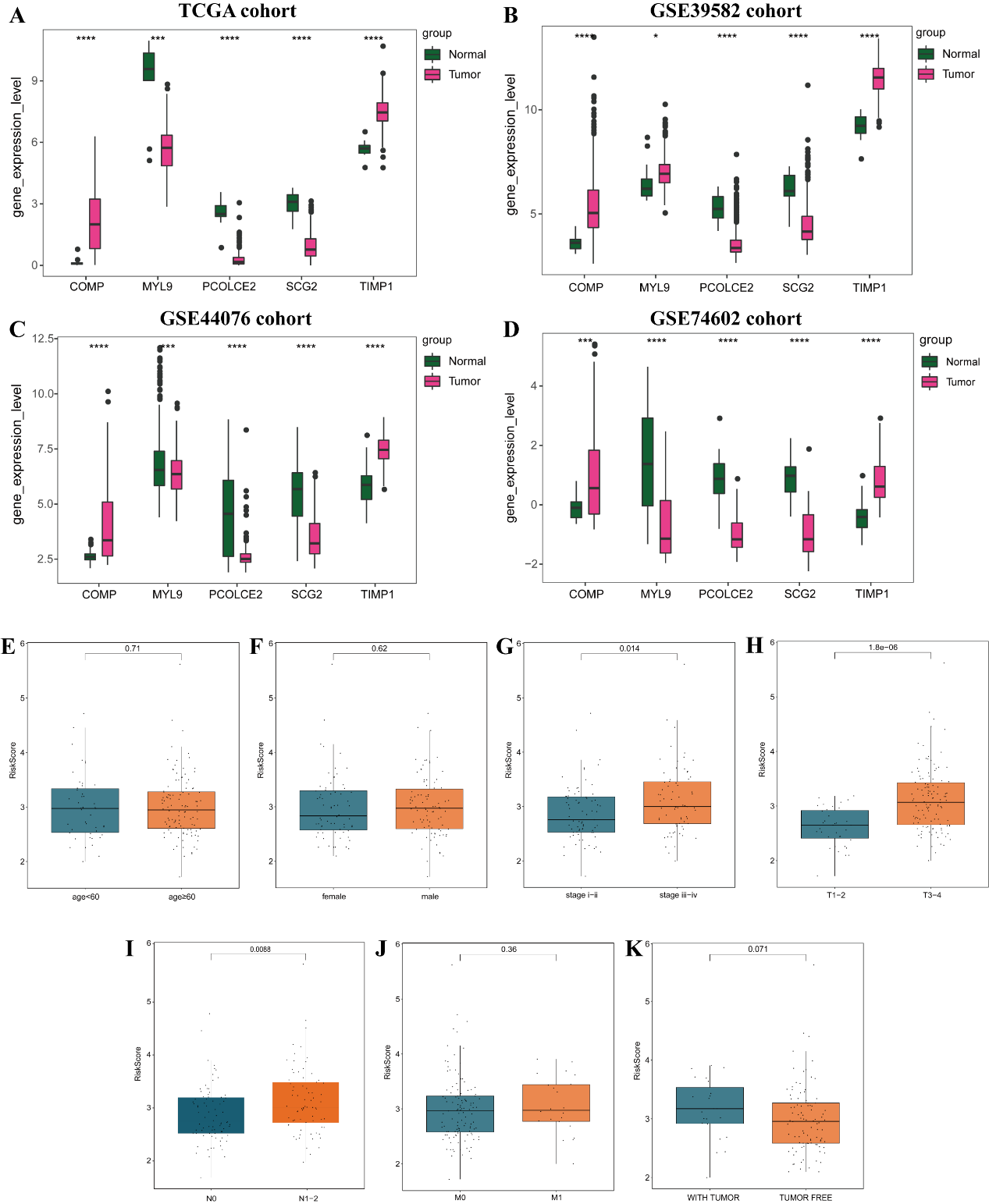
**

**Supplementary Figure 4 Relationship between the risk score and clinical characteristics of COAD.** Expression levels of five-EMTRG prognostic signature in (A) TCGA, (B) GSE39582, (C) GSE44076 and (D) GSE74602 cohort. TCGA cohort differences in risk scores across clinical characteristics including (E) age, (F) gender, (G) tumor stage, (H) pathologic T, (I) pathologic N, (J) pathologic M, and (K) cancer status.

**
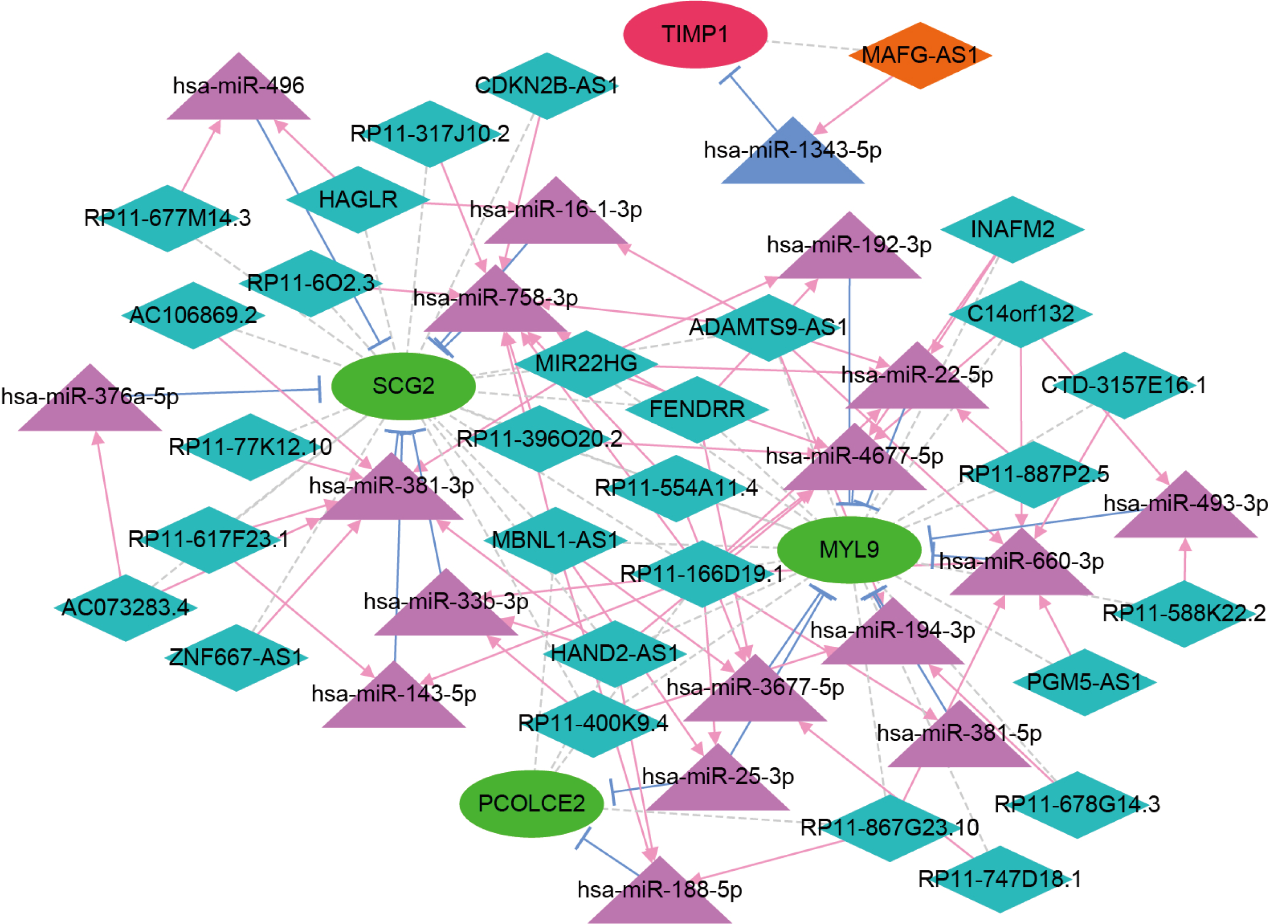
**

**Supplementary Figure 5 Construction of a Five-EMTRG Prognostic Signature related ceRNA network.** Red circles represent up-regulated EMTRG and green circles represent down-regulated EMTRG. Yellow diamond represents up-regulated lncRNA and the cyan diamond represents down-regulated lncRNA. Purple triangles indicate up-regulated miRNAs and blue triangles indicate down-regulated miRNAs. Pink arrow linkage represents lncRNA competitive binding miRNA, blue T-shaped linkage represents miRNA-mRNA regulatory relationship, and gray dashed line represents lncRNA-mRNA co-expression relationship.


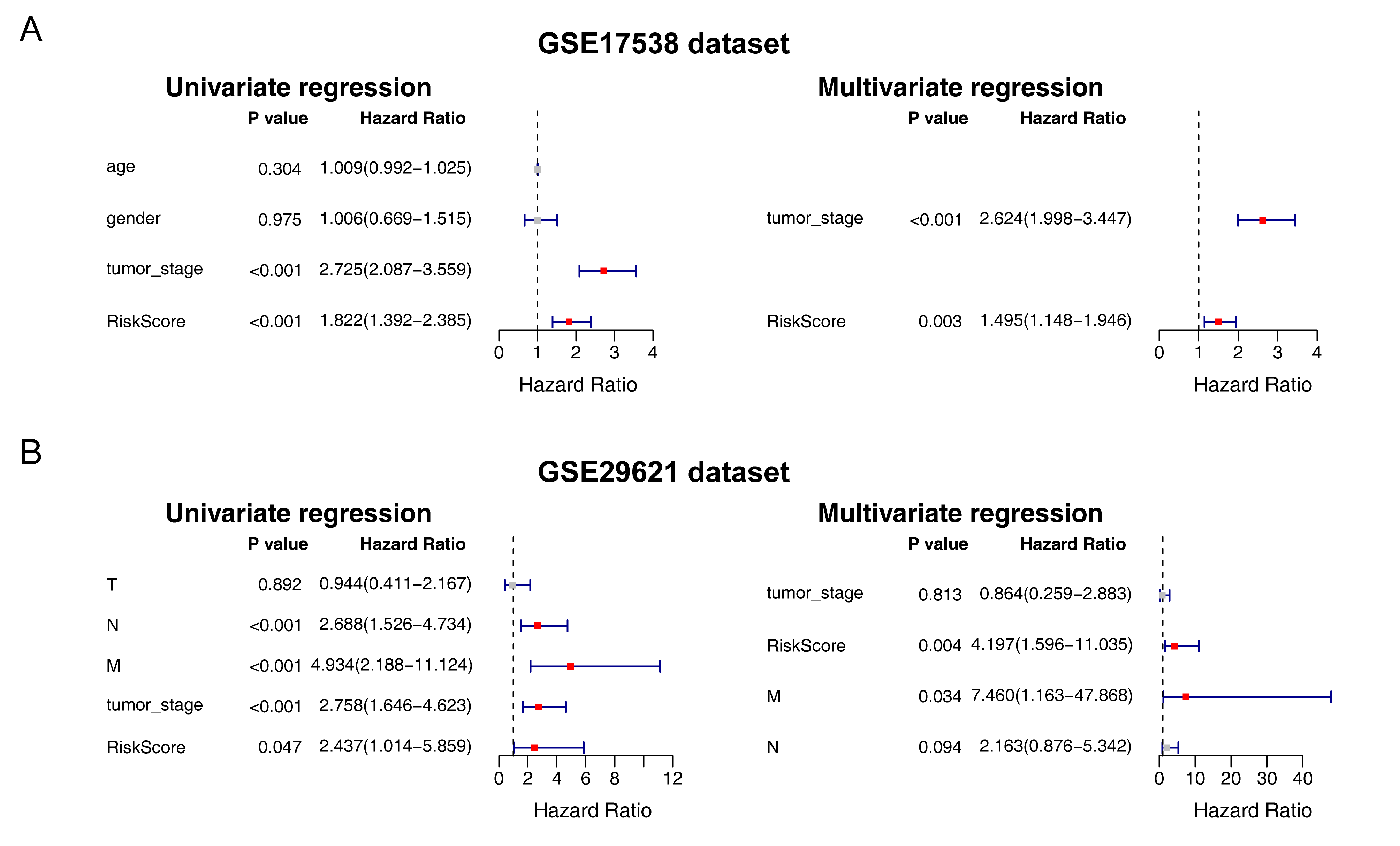


**Supplementary Figure 6. Univariate and multivariate Cox regression analyses.** (A) Univariate and multivariate Cox regression analyses of GSE17538 dataset. (B) Univariate and multivariate Cox regression analyses of GSE29621 dataset.
